# Case report: A case report and literature review about Pathological transformation of lung adenosquamous cell carcinoma

**DOI:** 10.3389/fonc.2022.1029679

**Published:** 2022-10-18

**Authors:** Liang Ge-ge, Geng Cuicui, Li Leiqiang, Tian Yongcang, Ma Jiangang, Ou Yiwen, Sun Li-zhe

**Affiliations:** Department of Oncology, The Second Affiliated Hospital of Shaanxi University of Traditional Chinese Medicine, Xianyang, China

**Keywords:** cardiac metastasis, lung cancer, pathological transformation, case report, EGFR-TKI

## Abstract

**Background:**

Lung adenosquamous carcinoma is a relatively rare pathological type in lung cancer. The incidence of gene mutation is lower than that of lung adenocarcinoma. However, the cases of pathological transformation after targeted treatment of EGFR gene mutation are more rare.

**Case introduction:**

A 55 year old female was diagnosed with lung cancer and underwent surgical treatment.The pathology suggested adenosquamous cell carcinoma. Genetic test was EGFR-L858R. After surgery, she was treated with gefitinib targeted therapy. After 2 years of surgery, she developed brain metastasis. surgery was performed again. The pathology suggested squamous cell carcinoma. She continued to take gefitinib targeted therapy orally. After one month later since brain metastasis, she was found to have heart cavity metastasis and surgery was performed for the third time. Besides, the pathology suggested adenosquamous cell carcinoma. Genetic test was EGFR-p E746_ A750del, T790M (-), and we replaced with the second-generation EGFR-TKI afatinib targeted therapy. Up to now, no recurrence or metastasis has been found.

**Conclusion:**

We now report a rare case of lung adenosquamous carcinoma with pathological transformation during targeted therapy, which is intended to provide therapeutic ideas for the treatment of lung adenosquamous carcinoma in clinical practice. In addition, we reviewed previously reported tumor heterogeneity in the literature.

## Introduction

Lung cancer is one of the most common malignant tumors, has distinct pathologic subtypes with diverse biological characteristics. Adenosquamous carcinoma is a rare subtype, and its malignancy and prognosis are higher and worse than those of adenocarcinoma or squamous cell carcinoma. As an important factor affecting prognosis, metastasis plays an important role in tumor staging and treatment strategies. In terms of treatment, surgery, chemotherapy and radiotherapy are still the main methods. Some patients with epidermal growth factor receptor (EGFR) gene mutations can benefit from tyrosine kinase inhibitor (TKI) treatment (1). This study collected the pathological transformation of metastatic lesions in a case of lung adenosquamous cell carcinoma after TKI treatment.

## Case description

**Figure 1:**
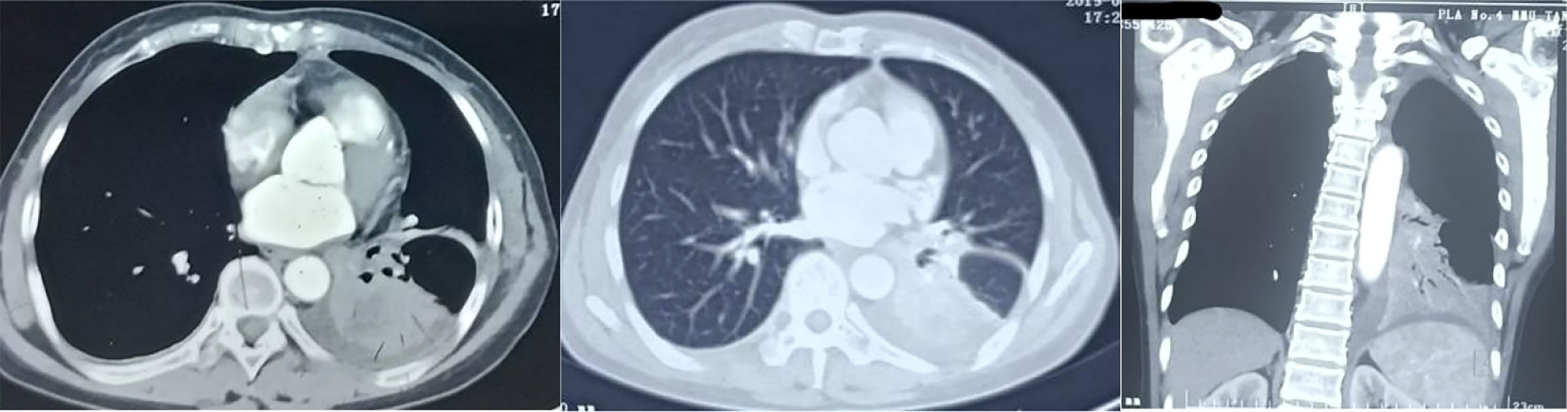
Chest CT.

**Figure 2:**
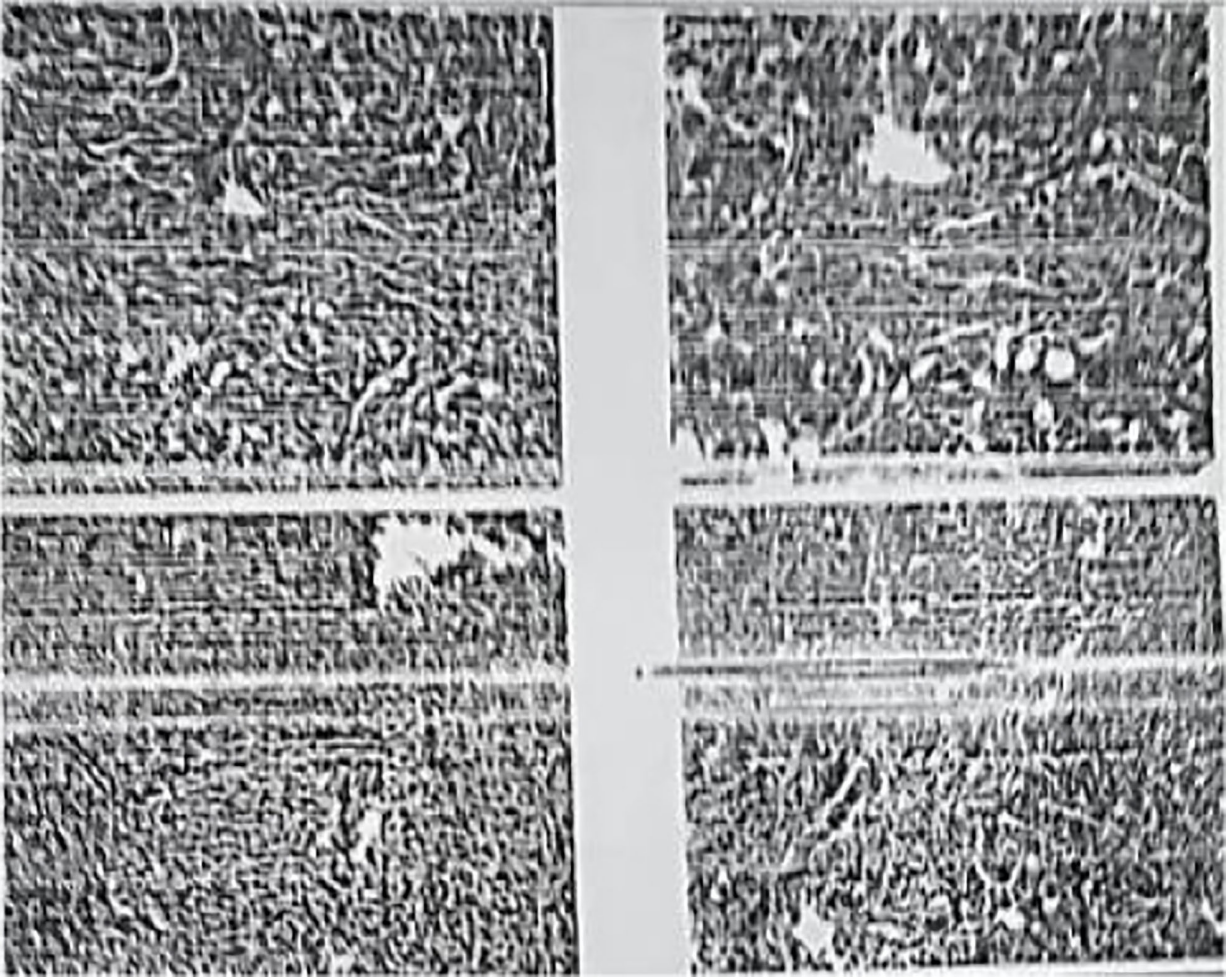
Postoperative pathology (Lung).

The patient, a 55 year old female, no previous disease history, no bad habits of personal history. And no history of tumor, infection or genetic disease in the family, visited our hospital on July 17, 2021 with the chief complaint of “two and a half years after lung cancer surgery and one month after brain metastasis surgery”. On January 13, 2019, she went to Tangdu Hospital of the Fourth Military Medical University with “intermittent cough and expectoration for more than 20 days, and lung cancer was diagnosed for 2 days”. Before admission, she performed chest CT in the First Affiliated Hospital of Xi’an Jiaotong University (**Figure 1**) showed left hilar space occupying lesions, more central lung cancer, obstructive pneumonia and left lower lobe atelectasis were considered. After admission, blood routine test, liver and kidney function, electrolyte, blood glucose, blood coagulation and infection were checked. Bronchoscopic biopsy was that (lower lobe of left lung) the submitted lung tissue was chronic inflammation with fibrous tissue hyperplasia, and a few atypical cells were found locally. The histological characteristics suggested that the possibility of canceration in a few washbars could not be completely excluded. PET/CT:1. Left lower lobe space occupying, left hilar enlarged lymph nodes, increased glucose metabolism, consistent with “left lung cancer” with left hilar lymph node metastasis; 2. No obvious abnormal metabolic signs are found in other parts. The clinical stage was cT2bN1M0, phase IIB. There was no surgical contraindication. On January 21, 2019, under general anesthesia, thoracoscopic radical resection of lung cancer (left lower lobe resection+regional lymph node dissection) was performed successfully. Postoperative pathology (**Figure 2**): specimens of left lower lobe resection: (left) central adenosquamous carcinoma of the lung (lower) lobe, mainly squamous cell carcinoma, with no invasion of the pleura; No cancer tissue was found in the bronchial stump; Metastatic cancer was found in hilar lymph nodes, and no cancer tissue was found in para bronchial lymph nodes and lymph nodes in groups 5, 7 and 9/11; In addition, 6 groups of lymph nodes were sent as fibrous adipose tissue, and no cancer tissue was found. Gene detection showed EGFR-L858R and TP53 mutations. After surgery, gefitinib(250mg Qd) was taken orally for targeted treatment, and the condition was stable without discomfort.

**Figure 3:**
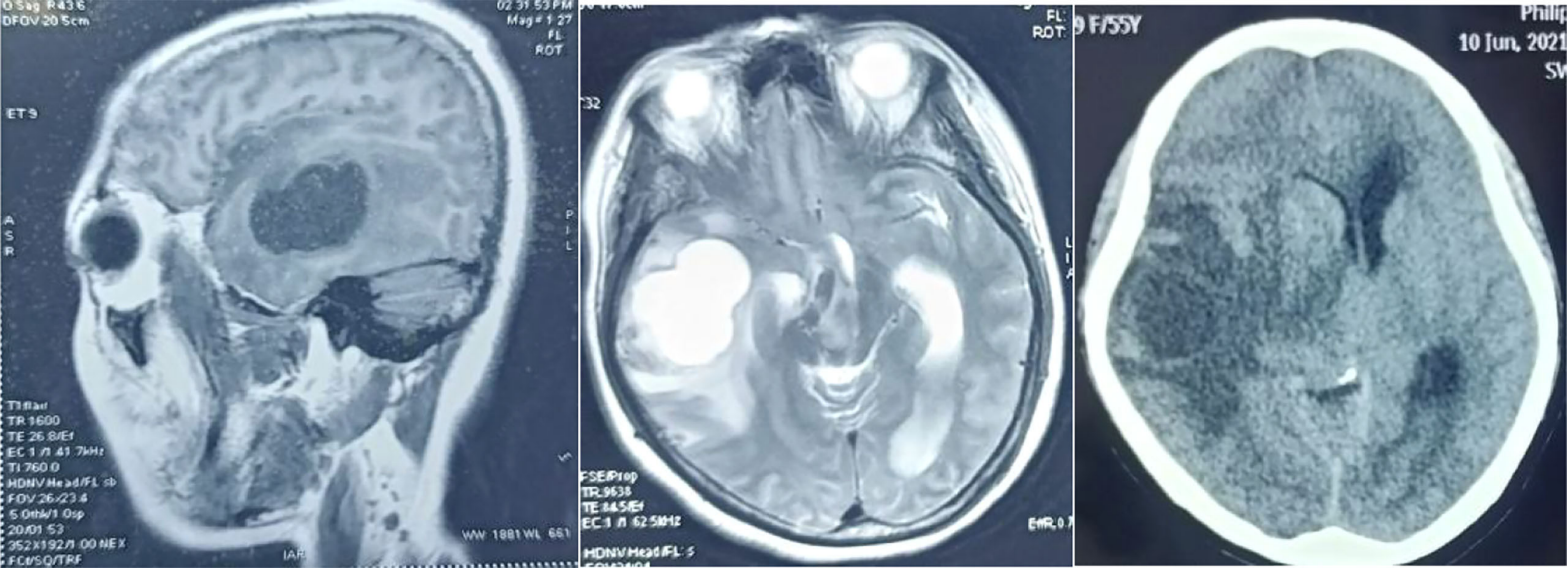
Head MRI.

**Figure 4:**
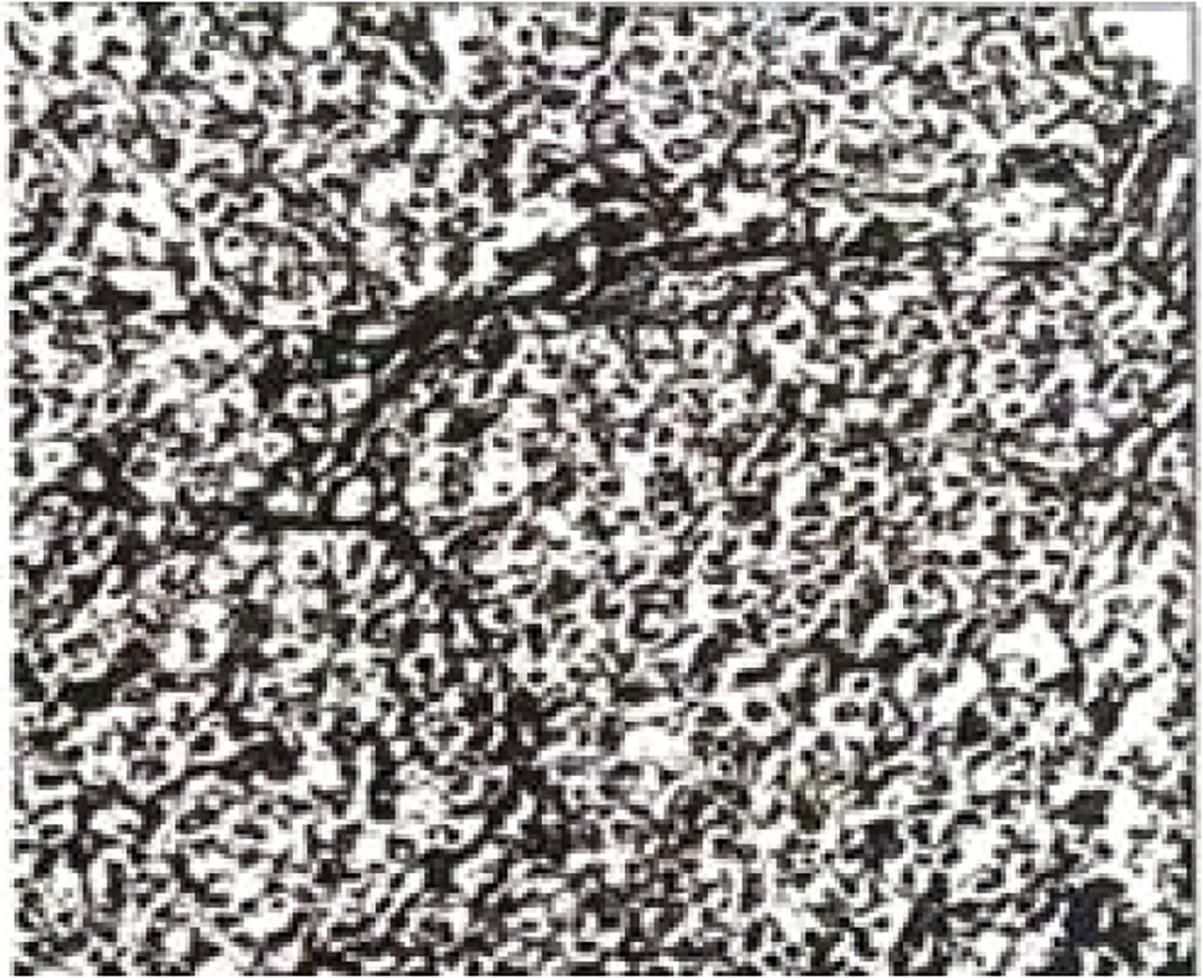
Postoperative pathology (head).

On June 8, 2021, the patient presented with intermittent headache, nausea, vomiting, and hallucination. She was admitted to the 215 Hospital of Shaanxi Nuclear Industry. Physical examination: lethargy, lack of cooperation, no abnormality was found in the heart, lungs, and abdomen. The muscle strength of the left lower limb was Grade 4, and that of the other limbs was Grade 5. Muscle tension was normal. The superficial and deep reflexes were normal, and the pathological signs were negative. The ranges of blood routine test, liver and kidney function, electrolyte, blood sugar, blood coagulation and infection were normal. Head MRI: The right temporal lobe had a cystic solid space occupying lesion. The lesion was mainly cystic, surrounded by large areas of edema, involving the right basal ganglia, thalamus and brain stem, and brain hernia. It was recommended that MR enhancement be further examined. Enhanced head MRI (**Figure 3**): cystic space occupying lesion in the right temporal lobe, with significantly enhanced uneven rosette wall, surrounding moderate brain edema area, the midline was compressed and moved to the left about 1.1cm. Considering malignant tumor, the possibility of brain metastasis was high. Chest and abdomen CT: 1. Changes after left lower lobe resection, left upper lobe and right middle and lower lobe cord foci; 2. Pleural hypertrophy on both sides with a small amount of pleural effusion; 3. Low density focus of liver, considering hemangioma. Dehydration and lowering intracranial pressure were given. Subsequently, the family members agreed to the surgical treatment. On June 13, 2021, supratentorial craniotomy was performed to remove the right frontal lobe space occupying lesion. The surgical procedure was Successful. Postoperative pathology (**Figure 4**): (right temporal part) poorly metastatic carcinoma with massive hemorrhage and necrosis, which combined with immunophenotype conforms to poorly differentiated squamous cell carcinoma. After the operation, gefitinib (250mg Qd)was taken orally for targeted treatment without complaints of discomfort.

**Figure 5:**
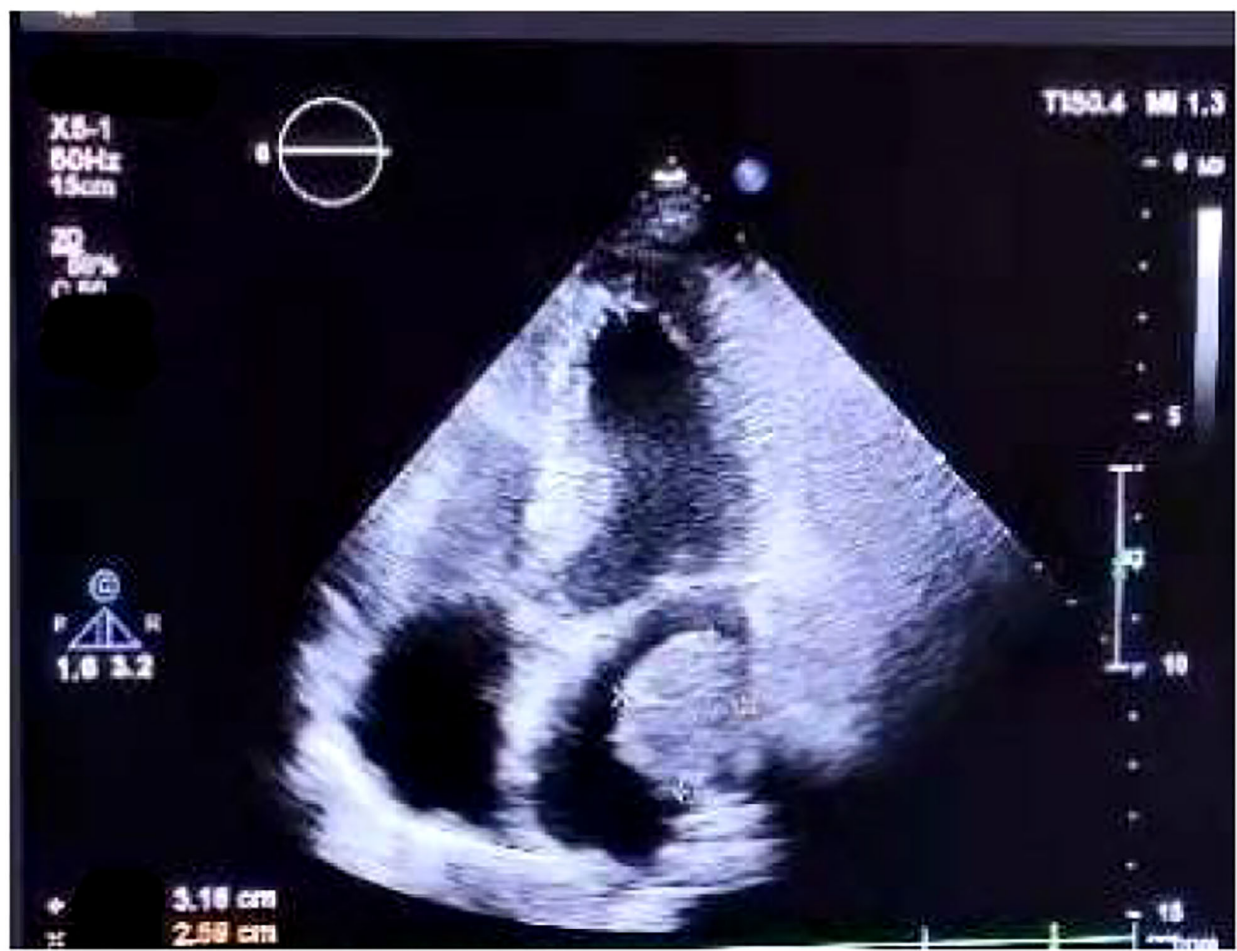
Echocardiography.

**Figure 6:**
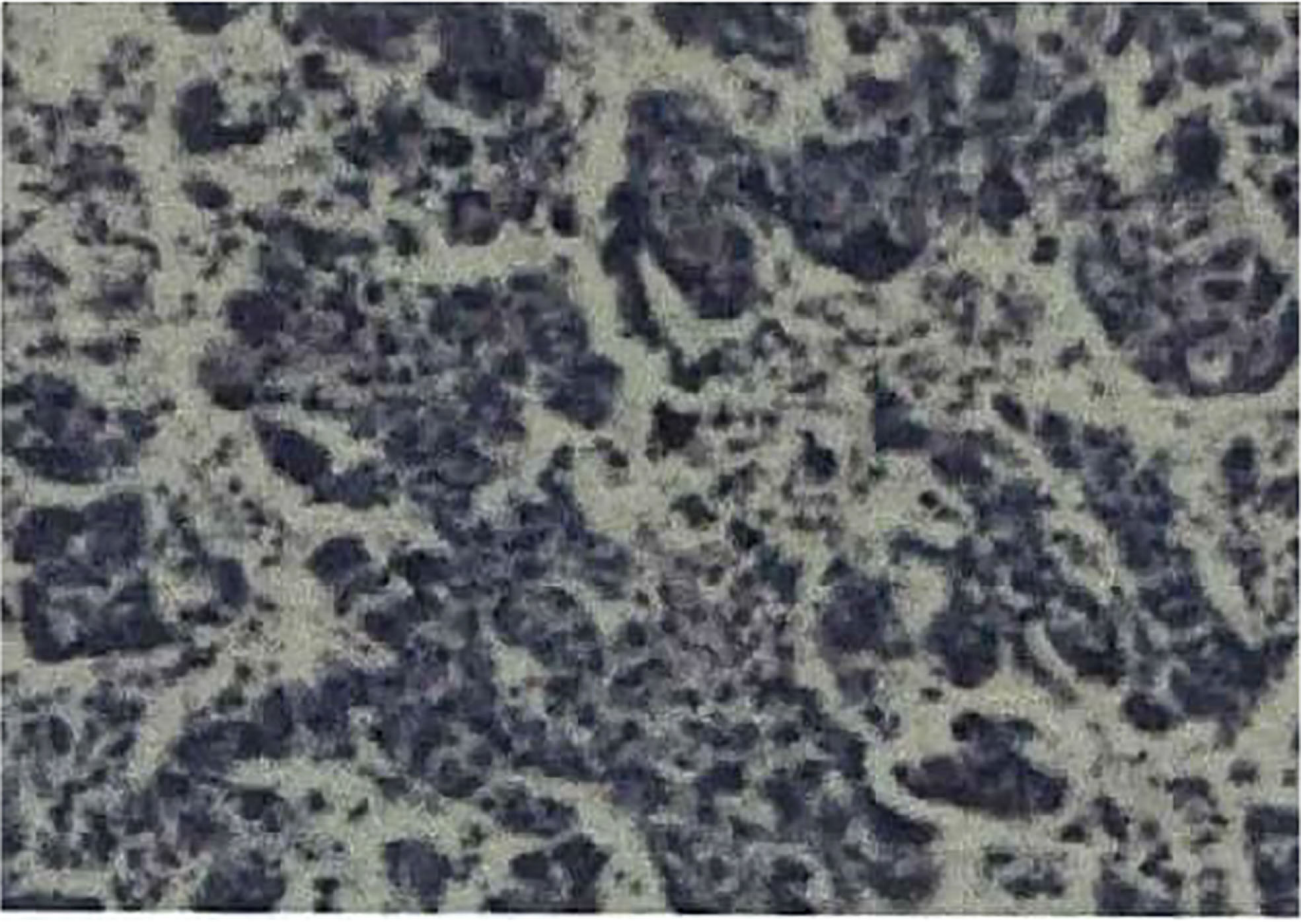
Postoperative pathology (Heart)HE × 100.

**Figure 7:**
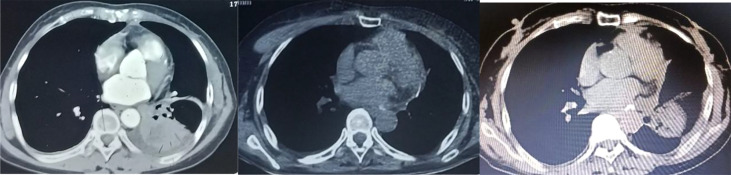
Chest CT contrast (From left to right:2019.1-2021.6-2022.4).

On July 17, 2021, she came our department for continuing treatment with occasional headache, acupuncture-like, which can relief itself. Physical examination: The general condition was good, no abnormality in the heart, lungs and abdomen, and no swelling of both lower extremities. And echocardiography (outside hospital on 2021-7-15): hypoechoic left atrial cavity, consider: space-occupying lesion. After admission, the blood routine, urine routine, liver and kidney function, tumor markers were within the normal range, and the electrocardiogram was generally normal. Cranial MRI,Chest and whole abdominal CT: no recurrence and metastasis were found, and repeated echocardiography: medium echogenic mass in the left atrium (about 1.8cm×2.9cm in size), see **Figure 5**. The patient developed chest tightness and palpitation, and the electrocardiogram showed atrial fibrillation. After symptomatic treatment, sinus rhythm was restored. On 2021-07-23, left atrial tumor resection was performed under general anesthesia and low-temperature cardiopulmonary bypass. Postoperative pathology (**Figure 6**) suggests (left atrium) malignancy, tending to poorly differentiated adenosquamous carcinomamalignant tumors,. Gene detection: EGFR-p.E746_A750del, T790M(-).Therapy was replaced with afatinib(40mg Qd). Since the operation, the general state is good, and no recurrence (**Figure 7**).

## Discuss

According to the current epidemiological survey, the incidence and mortality of lung cancer are now at the forefront (2). Lung adenosquamous carcinoma (ASC) is a rare subtype of non-small cell lung cancer, accounting for only 0.4% to 4% of lung cancer patients (3, 4), but research on changes in the spectrum of rare lung tumors suggests: The proportion of adenosquamous carcinoma increased slowly, from 0.84% in 2004 to 1.25% in 2015, and surpassed large cell carcinoma after 2011, becoming the most common tumor among rare lung cancers (5). Its composition includes lung adenocarcinoma (LUAD) and lung squamous cell carcinoma (LUSC) components, and in terms of morphological characteristics, it has the characteristics of classic LUAD and LUSC, but the behavior is more aggressive. The diagnostic criteria is that any component of squamous cell carcinoma and adenocarcinoma accounts for at least 10% of all tumors. Therefore, it is easy to miss diagnosis due to the limitations of biopsy specimens in clinical work.

The different pathology of metastatic lesions and primary lesions in cases reflects a major feature of tumors—heterogeneity. Tumor heterogeneity exists between patients, within tumors, and between tumors, and between-tumor heterogeneity is considered to be the diversity between the primary tumor and its metastases (6–8), which is common in patients with malignant tumors. Moreover,it plays a key role in diagnosis and treatment.

In lung cancer, its heterogeneity is related to different genetic, epigenetic, and non-genetic mechanisms (9, 10). Lung cancer development represents the initiation of a multistep process involving genetic alterations, the most important feature of which is extensive genomic aberrations, including abnormalities, gain and loss of large chromosomal regions, gene rearrangements, copy number gain, amplification (11), and the essence of these molecular-level abnormalities reflects genomic instability. Regardless of risk factors such as tumor stage, age, and gender, genomic instability is often associated with poor prognosis (12, 13), and even genomic diversity contributes to the adaptation of cancer cell populations in the tumor microenvironment, leading to tumor progression and poor prognosis.

Mutant allele-specific imbalance (MASI) is another genetic mechanism that promotes heterogeneity and affects tumor initiation, progression, metastasis, prognosis, and potential therapeutic response. MASI is common in some major oncogenes, such as EGFR, KRAS, PIK3CA, and BRAF (14), and epigenetic modifications induce variability in gene expression, determining a significant diversity according to previous studies. Furthermore, variable pressures in the lung tumor environment can generate inter-and intra-tumor heterogeneity, which affects sensitivity to targeted therapy and immunotherapy response (15).

Given the heterogeneity of lung cancer, the genomic background of ASC has been still poorly understood. They found that there were not only TP53 mutations, but also EGFR, met and BRAF mutations in adenosquamous cell carcinoma in the project by Arthur Krause et al, who applied whole genome exon sequencing technology to interpret the clonal relationship between adenocarcinoma and squamous cell carcinoma in ASC (16). According to statistics, EGFR mutations were at least as common in ASC as in classical LUAD, with mutation rates of 15% ~ 50%, while only about 2% mutations were reported in LUSC (17–19).

With the advent of new drugs, targeted therapy has become a hotspot in the research and treatment of lung cancer. The most representative is targeted therapy for EGFR mutations in lung denocarcinoma, studies such as LUX-LUNG 7, ARCHER 1050, FLAURA and EVOLUTION (20–25) have confirmed significant benefits for those with EGFR mutations, whether in the first-line, second-line or postoperative adjuvant therapy; Iwanaga et al (26, 27) report that ASC patients after surgery with EGFR sensitive mutation receive gefitinib as a second-line treatment,and achieve a 3-year progression free survival. A study published in the journal ANNALS OF ONCOLOGY further confirmed that EGFR-TKI is effective in treating ASC patients with EGFR mutation positive (1).For oligometastatic lesions during the period of first-line EGFR-TKI treatment, EGFR-TKI treatment can be continued and combined with local treatment. For patients of lung cancer with cardiac metastasis, through literature searching, local treatment, such as surgical resection or radiotherapy, has also got good outcome (28–31).

When lung cancer was found in this patient, surgery was performed. Pathology suggested adenosquamous cell carcinoma. After surgery, gefitinib targeted therapy was performed. Brain metastasis occurred after 2 years, and surgery was performed again. Pathology suggested squamous cell carcinoma. The number of materials was sufficient and the accuracy was high. ASC was basically ruled out. After TKI targeted drug treatment, the patient induced ASC to transform into squamous cell carcinoma. It was speculated that TKI had therapeutic advantages on adenocarcinoma components, which directly leaded to the withering of adenocarcinoma component cells, while squamous cell carcinoma cells gain a quantitative advantage. Cardiac cavity metastasis was found after 1 month of brain metastasis. Postoperative pathology showed adenosquamous cell carcinoma, suggesting the existence of drug resistance and heterogeneity between metastatic lesions. It was replaced with second-generation EGFR-TKI afatinib targeted therapy.

## Conclusion

ASC can have pathological transformation during the treatment process, which should be fully considered when making treatment plans; ASC patients with EGFR sensitive mutations can benefit from TKI treatment. Routine EGFR gene testing is recommended for ASC patients; When disease progression occurs in the established treatment plan, the existence of secondary biopsy and gene testing is necessary. We has followed the paitient all the time since she accepted the heart surgery and want to provide treatment ideas for ASC patients by this case. The only inadequacy is that the early treatment of patients is not carried out in our hospital, and some data in particular of figures are not provided clearly.

## Data availability statement

The datasets presented in this article are not readily available because of ethical/privacy restrictions. Requests to access the datasets should be directed to the corresponding author.

## Ethics statement

The studies involving human participants were reviewed and approved by the Ethics Committee of Drug Clinical Trials of the Second Affiliated Hospital of Shaanxi University of Traditional Chinese Medicine. The patients/participants provided their written informed consent to participate in this study. Written informed consent was obtained from the individual(s) for the publication of any potentially identifiable images or data included in this article.

## Author contributions

LG-G was responsible for the clinical design and conceptualization. MJ, OY and TY were involved in the acquisition of the clinical data. SL and GC conducted the clinical diagnosis. LL analyzed and interpreted the data. LG-G and GC wrote the manuscript. All authors contributed to the article and approved the submitted version.

## Funding

This study was funded by a grant from Natural Science Foundation of Shaanxi Province(Grant Number: S2020-JC-YB-0123).

## Conflict of interest

The authors declare that the research was conducted in the absence of any commercial or financial relationships that could be construed as a potential conflict of interest.

## Publisher’s note

All claims expressed in this article are solely those of the authors and do not necessarily represent those of their affiliated organizations, or those of the publisher, the editors and the reviewers. Any product that may be evaluated in this article, or claim that may be made by its manufacturer, is not guaranteed or endorsed by the publisher.
